# Human papillomavirus type 16 genomic variation in women with subsequent in situ or invasive cervical cancer: prospective population-based study

**DOI:** 10.1038/s41416-018-0311-7

**Published:** 2018-10-22

**Authors:** Laila Sara Arroyo-Mühr, Camilla Lagheden, Emilie Hultin, Carina Eklund, Hans-Olov Adami, Joakim Dillner, Karin Sundström

**Affiliations:** 1 Department of Laboratory Medicine, Karolinska Institutet, Stockholm, Sweden; 2 Department of Medical Epidemiology and Biostatistics, Karolinska Institutet, Stockholm, Sweden; 3 Clinical Effectiveness Research Group, Institute of Health and Society, University of Oslo, Oslo, Norway; 4 Center for Cervical Cancer Prevention, Karolinska University Laboratory, Karolinska University Hospital, Stockholm, Sweden

## Abstract

**Background:**

HPV genomic variation may be involved in viral carcinogenesis.

**Methods:**

In a national register-based nested case–control study, we retrieved archival smears from baseline cytologically normal women who later developed cancer in situ (CIS), squamous cervical cancer (SCC) or remained free of disease. These smears were previously HPV tested by PCR and HPV16 was the strongest risk factor. We now used the Illumina NextSeq platform to sequence HPV16 genomes in cervical smears from 242 women who later developed CIS/CIN3 (*n* *=* 134), SCC (*n* *=* 92) or remained healthy (*n* *=* 16).

**Results:**

The median sequence depth per sample was high (11,288×). For 218/242 samples (>90%), we covered ≥80% of the complete HPV16 genome with sequencing median depths of >200×. We identified a wide range of unique isolates and 147 novel SNPs across the 218 samples. Most women (97%) had HPV16 lineage A infection, with the sublineages being A1 (66.1%), A2 (28.9%) and A4 (1.8%), respectively. The least variable gene was the *E7* (3.4% variability), where 170/204 case women (83%) displayed a fully conserved sequence. There were no obvious differences by disease outcome (CIS or SCC).

**Conclusions:**

We found a high number of novel SNPs. The *E7* gene was hypovariable both among women later developing CIN3/CIS, and SCC, respectively.

## Background

The factors that favour progression of human papillomavirus (HPV) infection to cervical cancer are incompletely understood. Advances in next-generation sequencing nowadays allow deep, high-quality sequencing of entire viral genomes in HPV-positive samples from large cohorts, enabling assessment of the importance of viral genomics. HPV16 variant lineages have been implicated in cervical carcinogenesis. The definition of a variant lineage is that the L1 open reading frame differs by more than 1% but less than the 10% that would make it another HPV type^1^. A variant sublineage is defined as groups of sequences with 0.5–1.0% differences between genomes^2^. The four identified variants of HPV16 are divided into sublineages A1–3 (formerly termed European), A4 (Asian); B (African-1), C (African-2) and D1–3 (North-American, Asian-American) and have been associated with different cervical precancer and cancer risk^3^. Even within HPV16 variants, genetic polymorphisms may play a key role for infection persistence and oncogenic potential^3^.

Primer sets that amplify the whole 8-kilobase pair HPV16 genome have been described^4^. Conservation of the *E7* gene appears to be required for cervical carcinogenesis^5^. The remaining issues that need to be addressed are: (i) if thorough sequencing will reveal additional sequence variability (single-nucleotide polymorphisms (SNPs)), (ii) if variability is preferentially associated with invasive or in situ cancer and (iii) if variability can be detected already before development of disease in prospective studies. We therefore investigated viral gene variation in a prospective, population-based study of invasive and in situ cervical cancer, using the Illumina NextSeq sequencing platform that enables deep and large-scale sequencing.

## Materials and methods

### Study participants

The study design has been described previously^6^. In brief, all squamous cervical cancer (SCC) case women in Sweden during the period 1969–2002 were identified using the Swedish National Cervical Screening Register. We used the same register to draw a random sample of case women with carcinoma in situ (CIS; equivalent to CIN3). Using case–control sampling, we then identified one woman, matched on county, date of entry into cohort (all women in Sweden with a normal smear) (±3 months) and age at first normal smear (±1 year), as an individually matched control for each CIS and SCC case. We retrieved archival pre-diagnostic smears from the case women and matched smears from the control women. Histologic specimens from the identified cases were re-reviewed by a senior pathologist. All pre-diagnostic specimens were tested for HPV and the viral load determined by real-time PCR^7^.

The first smear from all single-infection HPV16-positive samples (*n* *=* 1250) that were found to have an HPV16 viral load >100 copies/µl (*n* *=* 439) were retrieved for sequencing. Overall, we used 242 samples from 242 different women with either CIS/CIN3 (146 samples: 134 cases and 12 controls) or SCC (96 samples: 92 cases and 4 controls).

### Extraction of DNA

The smears taken prior to the date of diagnosis of the case in each case–control pair had been requested from comprehensive archives. DNA was extracted from archival cervical conventional Pap smears, as previously described^6^. For our HPV16 sequencing, the HPV16-positive samples were subjected to an additional purification step which was performed using MagNa Pure LC Total Nucleic Acid Isolation Kit in accordance with the manufacturer's protocol (Roche Molecular Systems, Inc., Alameda, CA, USA).

### DNA amplification and primer pooling

The entire HPV16 genome (7906 bp) was amplified as 47 overlapping amplicons, ranging in size from 181 bp to 375 bp, as previously described^4^. Amplification primers were divided into five different reactions (Supplementary Table 1) to reduce occurrence of self-dimers and cross-primer dimers, and extracted DNA was amplified separately by all five PCR reactions for every sample. After PCR, amplification products were pooled together according to sample name, before library preparation. To control for contamination and accuracy, negative controls (Sigma water as well as HPV16-negative but human DNA-positive controls) were also amplified. Each PCR was performed with 5 µl DNA in 20 µl reaction containing 1x Qiagen Multiplex PCR Master Mix (Qiagen, Hilden, Germany) and 0.2 µM of each primer. A pre-heat of 95 °C for 15 min was followed by 45 cycles at 95 °C 30 s, 57 °C 90 s and 72 °C, 90 s, with a final extension at 72 °C for 10 min.

### Illumina library preparation

A quality analysis (Bioanalyzer, Agilent, Santa Clara, CA, USA) was performed to check DNA amplicon length prior to library preparation. A total of 255 libraries (242 samples, 9 PCR negative controls with Sigma water and 4 PCR negative controls containing human DNA) were prepared using the TruSeq Nano DNA Sample Preparation kit according to the user guide revision A (Illumina, San Diego, CA, USA) with the following modifications: as each sample consisted of approximately 200–400 bp long PCR products, the tagmentation, end-repair and size selection steps were omitted, and hence the library preparation started with adenylation of 3’-ends. We used 75 ng of PCR product as input in a volume of 17.5 μl of resuspension buffer and 2 adaptor indexed primers were ligated to each sample.

All individual libraries were validated, normalised to 2 nM and pooled in different pools. Each pool contained approximately 48 libraries (including specimens and negative controls) and was denatured and diluted, resulting in a 1.8 pM DNA solution. All library pools were sequenced paired-end 151 + 151 cycles once, using the NextSeq 500 instrument and NextSeq 500 High Output reagent kit (Illumina) as described in the user guides Denature and Dilute Libraries Guide v02 for the NextSeq System, NextSeq 500 kit Reference Guide revision F and NextSeq 500 System Guide v02.

### Sequence analyses

We used indices included in the Illumina adaptors to assign raw sequence reads obtained from the NextSeq 500 (Illumina) platform to the originating samples. Reads were quality and adaptor trimmed with Trimmomatic^8^. All reads with a read length below 150 base pairs (bp) were discarded for further analysis. We aligned 150 bp long quality reads to a modified HPV16REF (human papillomavirus 16 reference sequence from the The PapillomaVirus Episteme, 7906 bp) using NextGenMap^9^. We considered only paired-end reads where both reads mapped to the genome, with the correct orientation and distance, with >90% identity over 75% of their length as valid and further analysed.

As the HPV genome is circular, the reference genome was modified by adding to the end (after position 7906), the first 258 nucleotides in order to not lose coverage of amplicons 46 and 47, which start at the end of the genome and end at the beginning.

To filter out human reads, HPV16 mapped reads from the first 48 samples were screened against the human reference genome hg19 using NextGenMap^9^ with same parameters (>90% identity over 75% of their length). No reads mapped to human sequences, and therefore filtering of human reads was omitted from the bioinformatics pipeline.

Resulting BAM files were merged according to sample names, processed through a quality control and left aligned using the GATK version 3.8, LeftAlignIndels Module.

GATK DepthofCoverage was used to perform coverage analysis and generating coverage summary plots. Each nucleotide position had to have more than 5 reads to be considered as “covered”. Amplicons primers were trimmed from aligned reads using BamUtil Trimbam (http://genome.sph.umich.edu/wiki/BamUtil:_trimBam), trimming 32 bases from the 5′-end.

### HPV16 variant calling

The HPV16 genome was genotyped by GATK HaplotypeCaller Version 3.8. SNP and indel calls were made and hard filtered, following GATK Best Practices. All variant calls met the following conditions: QualbyDepth <2.0, FisherStrand >60.0, Root Mean Square Mapping Quality <40.0, Mapping Quality Rank Sum Test <−12.5 and Read Pos Rank Sum Test <−8.0 to avoid strand biases, inflation when there was deep coverage, false calls at the end of the reads and low-quality variant calls. All identified nucleotide variants were manually inspected and were only considered as true variants if the call showed at least 100 reads. For each sample, a whole-genome sequence fasta file was generated. All identified nucleotide variants were annotated in a database including the HPV16 gene or region and amino-acid changes.

### HPV16 variant lineage assignment

HPV16 variant lineage assignment was based on the maximum likelihood tree topology constructed using MEGA 7^10^, including 10 HPV16 European and non-European variant lineage reference sequences^1^, and lineage assignments were confirmed with SNP patterns. Variant lineage assignment was performed for all specimens excluding those with poor read depth (<200 median depth) and/or low genome coverage (<80% genome coverage).

### Identification of novel SNPs

All sequences with SNPs were blasted against HPV16 sequences deposited in GenBank (both complete and incomplete genomes, deposited until 18 August 2018), and those polymorphisms not reported in any deposited sequence were considered as “novel”.

### Statistical analyses

Descriptive statistics on viral mutations were presented along with 95% confidence intervals (CIs) and significance testing carried out using chi-square tests for differences in proportions between categories. All tests were two-sided and a *p* value of <0.05 was considered statistically significant.

The study was approved by the Regional Ethical Review Board of Stockholm which determined that informed consent from the participants was not required.

## Results

### HPV genome coverage

The median HPV16 viral load for all samples originally included was 496 copies/µl (range 103 copies/µl to 25,950 copies/µl). Overall, Illumina sequencing generated high-quality sequencing data at median depths of 200× to over 2000×, that covered 80 to 100% of the HPV16 genome in a single sequencing run (Table 1). A total of 218/242 specimens (90.1%) covered >80% of HPV16 complete genome (7906 bp) with sequencing median depths of >200×, with 135 samples (55.8%) covering 100% of the complete genome with sequencing median depths of >2000×. The median sequence depth per sample was high (11,288×).

**Table 1:** Sequence depth across genome coverage in 242 cervical samples

Sequence depth	HPV16 genome coverage			
	80%	85%	90%	95%
>200×	218	215	206	191
>500×	214	212	203	191
>1000x	209	208	199	189
>2000x	200	200	192	185

Summary statistics for samples that exceeded 80, 85, 90 and 95% sequence coverage at median depths greater than 200×, 500×, 1000× and 2000×. Each nucleotide position had to have more than 5 reads in order to be considered as covered

To consider a position in the genome as “covered”, a sequence depth of more than 5 reads was considered as mandatory. In one viral region (nt 5314–5404) 80–89/242 specimens (33.1–36.8%) did not present more than 5 reads per position. This 91 bp region is located within the HPV16 L2 gene, accounting for 1.2% of the viral genome and corresponded to amplicon 32 (non-overlapping part). Water controls and HPV16-negative but human DNA-positive controls had no HPV16 sequencing reads.

### Study participants

The median age at smear taking was 29 years for women with subsequent CIN3, and 39 years for women with subsequent SCC, with a median time from smear to diagnosis of 1.4 years and 2.1 years, respectively. The control women had a median age of 26 years at their smear (Table 2).

**Table 2:** Basic characteristics of the study participants

	*n*	Age at smear median (range)	Time to diagnosis median (range)	Age at diagnosis median (range)
CIN3	128 (58.7%)	29.3 (18.7–62.5)	1.4 (0.14–12.1)	31.8 (20.0–64.1)
SCC	76 (34.9%)	39.2 (19.4–68.0)	2.1 (0–19.5)	43.0 (25.9–75.5)
Controls	14 (6.4%)	25.7 (19.1–47.9)	–	–
TOTAL	218 (100%)	30.8 (18.7–68.0)	1.74 (0–19.5)	34.0 (20–75.5)

Age at smear (years), time between smear and diagnosis for the cases (years) and age at diagnosis for the cases (years)

### Variant lineages

We determined HPV16 variant lineage assignment based on a phylogenetic maximum likelihood tree for all 218 women, after excluding samples with poor median read depth (<200×) and/or low genome coverage (<80%) (Table 3). The 211 out of 218 women (96.8%) in our study had HPV16 variant lineage A infection, corresponding to 144 (66.1%) A1, 63 (28.9%) A2 and 4 (1.8%) A4 sublineages. We found 7/218 (3.2%) women with an HPV16 non-European variant lineage infection. Lineage C was detected in 1/218 (0.5%) women and lineage D in 6/218 (2.8%) women, corresponding to 1 woman classified as sublineage D1, 1 woman classified as sublineage D2 and 4 women classified as sublineage D3. Lineage B was not found in any participant. Lineage A thus dominated cases of both CIN3 and SCC, with no difference between lineages by phenotype (*p* = 0.27).

**Table 3:** HPV16 variants and subvariant lineages by diagnosis

		Variant sublineage assignation							
		*n* (%)							
	*n*	A1	A2	A4	D1	D2	D3	C	N/A
CIN 3	134	80	42	3	0	0	2	1	6
	(55.4)	(59.7)	(31.3)	(2.2)	0	0	(1.5)	(0.7)	(4.5)
SCC	92	52	19	1	1	1	2	0	16
	(38.0)	(56.5)	(20.7)	(1.1)	(1.1)	(1.1)	(2.2)	0	(17.4)
Control	16	12	2	0	0	0	0	0	2
	(6.6)	(75.0)	(12.5)	0	0	0	0	0	(12.5)
TOTAL	242	144	63	4	1	1	4	1	24
	(100)	(59.5)	(26.0)	(1.7)	(0.4)	(0.4)	(1.7)	(0.4)	(9.9)

Number of variant (sub)lineages detected in 242 HPV16-positive specimens

*CIN3* cervical intraepithelial neoplasia grade 3, *N/A* number of specimens with poor read depth (<200 median depth) and/or low genome coverage (80% genome coverage) that were not analysed for variant assignation, *SCC* squamous cell carcinoma

### HPV16 SNPs and indels

We detected a total of 598 SNPs and 25 indels (insertions and deletions) across the HPV16 genome when analysing all 218 specimens. We detected 563/598 (94.1%) SNPs and 6/25 (24.0%) indels within the HPV genes and upstream regulatory region (URR; Table 4), while 35/598 (5.9%) SNPs and 19/25 (76.0%) indels were detected within the HPV region 4102–4236 bp (non-coding region between *E5* and *L2*). SNPs and indels occurring within the *E4* region were considered as synonymous/missense/nonsense considering both protein-coding genes *E2* and *E4*. Therefore, one SNP in one position, e.g., position 3362, might be considered as missense in *E2*, but as synonymous in *E4*. Considering SNPs in all protein-coding regions, we detected a total of 280/510 (54.9%) non-synonymous substitutions, 223/510 (43.7%) silent variations, 7/510 (1.4%) SNPs that translated into premature stop codons and 5 indels (Table 4).

**Table 4:** HPV16 SNPs and indels

					SNP			
Gene/feature	Size (bp)	Total SNPs	% Variable sites (95% CI)	No. indels	Silent	Missense	Startloss	Nonsense
*E6*	477	25	5.24 (3.24–7.24)	0	11	14	0	0
*E7*	297	10	3.37 (1.32–5.42)	0	6	4	0	0
*E1*	1950	117	6.00 (4.95–7.05)	1	46	67	0	4
*E2*	1098	81	7.38 (5.83–8.92)	2	20	61	0	0
*E4*	288	29	10.07 (6.59–13.54)	2	19	10	0	0
*E5*	252	24	9.52 (5.90–13.15)	0	11	12	0	1
*L2*	1422	134	9.42 (7.90–10.94)	0	60	73	0	1
*L1*	1596	90	5.64 (4.51–6.77)	0	50	39	0	1
URR	832	90	10.82 (8.71–12.93)	3	–	–	–	–

Summary of HPV16 SNPs and indels detected in 218 HPV16-positive specimens of high coverage

*CI* confidence interval, *indel* insertion/deletion, SNP single-nucleotide polymorphism, *URR* upstream regulatory region

Regions exhibiting greater variability were URR (10.8% variability), followed by *E4* (10.1%), *E5* (9.5%) and *L2* (9.4%), while more conservative regions included the *E7, E6* and *L1* genes (3.4%, 5.2% and 5.6% variability, respectively). The most variable gene was thus *E4* (10.07%, 95% CI 6.59–13.54%) and the least variable *E7* (3.37%, 95% CI 1.32–5.42%; *p* = 0.001, Table 4).

Isolates showed a median substitution of 15 SNPs when we compared their complete sequences to the reference genome HPV16REF (min: 1 SNP, max: 131 SNPs). The majority of SNPs (320/598, 53.5%) were detected in only one specimen each. In all, 224/598 SNPs (37.5%) were identified in 2 to 10 specimens each (1 to 5% of total specimens), 30/598 SNPs (5.0%) were detected in 11 to 21 specimens (5–10% of total specimens) and 24/598 SNPs (4.0%) were found in at least 20 specimens each (>10% of total 218 specimens).

When we stratified by diagnosis (CIS or SCC), 430 SNPs were detected in CIN3 cases, and 387 SNPs in SCCs (Table 5). *L2* and *E1* genes showed the highest numbers of nucleotide substitutions (98 and 80 SNPs for CIN3 cases, and 84 and 70 SNPs for SCC cases, respectively). *E7* was the most conserved gene with only 8 identified SNPs for CIN3 and 9 for SCC (Table 5). Most women (183/218; 83.9%) displayed an *E7* sequence which was identical to the reference genome, i.e., fully conserved. By case–control status, 170/204 case women and 13/14 control women displayed 0 SNPs (Table 6). These women all had lineage A1, i.e., the same lineage as the reference genome we used. We observed a maximum of 3 SNPs per sample in the *E7* gene, and these occurred in women who had an infection of non-European lineage. Women infected with variants A1–A3 had highly conserved *E7* sequences with up to only 1 SNP difference to the reference sequence in their *E7* sequence.

**Table 5:** HPV16 SNPs and indels detected according to diagnosis

			CIN3	SCC	CONTROL
		*n*	128	76	14
SNPs, I	*E6*	S, MS, NS, I	10, 7, 0, 1	4, 10, 0, 0	1, 1, 0, 0
		Total	17	14	2
	*E7*	S, MS, NS, I	6, 2, 0, 0	5, 4, 0, 0	1, 0, 0, 0
		Total	8	9	1
	*E1*	S, MS, NS, I	36, 41, 3, 0	30, 40, 0, 1	2, 5, 1, 1
		Total	80	70	8
	*E2*	S, MS, NS, I	17, 47, 0, 0	15, 37, 0, 0	3, 7, 0, 0
		Total	64	52	10
	*E4*	S, MS, NS, I	17, 8, 0, 0	10,6, 0, 0	3, 1, 0, 0
		Total	25	16	4
	*E5*	S, MS, NS, I	7, 7, 1, 0	9, 8, 0, 0	3, 2, 0, 0
		Total	15	17	5
	*L2*	S, MS, NS, I	48, 50, 0, 0	37, 47, 0, 0	11, 8, 1, 0
		Total	98	84	20
	*L1*	S, MS, NS, I	36, 22, 0, 0	36, 23, 0, 0	7, 5, 1, 0
		Total	58	59	13
	URR	SNPs, I	67, 3	63, 0	11, 0
Total	TOTAL SNPs		430	387	75
	TOTAL INDELS		21	18	7

Number of SNPs and indels detected in 218 HPV16-positive specimens

*CIN3* cervical intraepithelial neoplasia grade 3, *I* indel, insertion/deletion, *MS* missense substitution, *n* number of specimens, *NS* nonsense substitution (stop codon), *S* synonymous substitution, *SCC* squamous cell carcinoma, SNP single-nucleotide polymorphism

**Table 6:** Number of SNPs in the *E7* gene in the 218 study participants

	0 SNPs	1 SNP	2 SNPs	3 SNPs	TOTAL
CIN3	106	17	2	3	128
SCC	64	7	2	3	76
Controls	13	1	0	0	14
Total	183	25	4	6	218

*SNP* single-nucleotide polymorphism, *CIN3* cervical intraepithelial neoplasia grade 3, *SCC* squamous cell carcinoma

### Novel SNPs

We identified up to 147 novel SNPs (not reported in GenBank), including 26 SNPs located within non-coding regions, 26 silent substitutions, 86 non-synonymous nucleotide variations, 5 nonsense substitutions and 4 SNPs that were classified as both silent and non-synonymous, depending on the protein to translate (*E4* vs *E2 or L2 vs L1*).

For each of the 8 protein-coding genes, the proportion of non-synonymous nucleotide substitutions that were novel ranged from 6.9% to 25.6% of all SNPs. All SNPs classified as novel are listed in Supplementary Table 2.

### HPV16 isolates

We studied 135 samples with coverage at 100% to determine how many women had identical isolates to each other. In these 135 women, we detected up to 123 unique isolates. Out of 135 women, 114 (84.4%) displayed HPV16 isolates differing by at least one nucleotide to any other woman’s sample. Among the remaining 21/135 women, 6 identical pairs of isolates were shared by two women each (6 isolates, 12 women) and 3 identical isolates were shared by 3 women each (3 isolates, 9 women).

## Discussion

We here report HPV16 genomic variation in a population-based set of cervical smears from a stringently designed prospective study of women in the Swedish population who had a normal smear but subsequently developed cervical cancer in situ or invasive squamous cervical cancer (CIN3+). We report the hitherto greatest sequencing depth in the literature. We applied a strong epidemiological study design, a high-quality sequencing protocol and a stringent bioinformatical algorithm. We identified mainly variant lineage A-positive women with a large amount of SNPs, 24.6% of which were classified as novel, and confirmed that the *E7* was the HPV gene that exhibits the least variability across samples. We detected more non-synonymous variants (55%) than silent substitutions, and all SNPs were determined not to be the result of an HPV lineage coinfection (data not shown) by identifying putative heterozygous allele calls (HPV is monoploid) at nucleotide positions that distinguished between variant (sub)lineages.

In samples with 100% sequencing coverage, we found that the sequence diversity was extensive, with 84.4% of women infected with isolates differing from those of other women in the study. Our data show perfect concordance with previous studies^5^ that reported nearly exactly the same estimates of unique circulating isolates (84.5%).

Similar to an American study, we found a dominance of A1/A2 subvariants, but few women with other variants or subvariants, likely due to the relative ethnic homogeneity of the Swedish population. Our finding is also expected given the strong association between A1/A2 subvariants and squamous epithelial cervical lesions^3^. We included no women with glandular lesions, who may be more prone to display D2/D3 variants^3,11^. We used the reference genome HPV16REF from The PapillomaVirus Episteme, since the original reference clone has known sequencing errors^12^. Others have opted to use HPV16R as their reference clone^4^, but the difference between these two2 reference genomes (HPV16R and HPV16REF) is only one nucleotide variation (A2926G, which was detected in all our 218 specimens). No isolate has yet been identified with identical sequence to HPV16R, but one isolate (GenBank accession number NC001526) shares identical nucleotide sequence to HPV16REF. This isolate does not, however, have the same position numbering as the HPV16REF. Use of the same HPV reference genome and making the calls in the same positions is essential to facilitate sharing and comparing results with other authors^13^.

The HPV16 *E7* gene was recently found to exhibit hypovariability in a large sample of invasive cervical cancers and this hypovariability was posited as a requirement for cervical carcinogenesis^5^. Using greater sequencing depth, we can here confirm that the *E7* gene was by far the most conserved gene across CIN3+ samples, indeed with a maximum of only 1 SNP difference observed to the reference among women infected with subvariants A1–A3, and only up to 3 SNPs even in women infected with other subvariant lineages.

The strengths of this study include the population-based prospective design within a national cervical screening programme. This meant that the HPV16-positive samples we included are guaranteed to have a very high generalisability to the female population. Other strengths include the comprehensive genotyping procedure assuring HPV16 presence in the sample, and stringency of sequencing analyses and bioinformatical procedures. Indeed, even in these archival samples, originally far from optimised for DNA sequencing, we obtained excellent statistics on depth and coverage. We improved 3/4 poorly performing amplicons previously reported^4^ using a deeper throughput as well as by dividing amplification primers into five different reactions to reduce occurrence of self-dimers and cross-primer dimers. While certain positions from one set of primers (amplicon 32) did not show coverage for up to 36.8% samples (89/292), the other 3 amplicons (amplicons 24, 26 and 47) had all their positions covered in at least 249/292 (85.3%) of samples. With the very high sequencing depth, the risk for erroneous calling of genomic variation was minimised.

The chief limitation of our study was that due to few control women being positive for HPV16, meaningful analyses of risk associations comparing to healthy controls were not possible and inference on *E7* hypovariability in healthy women was limited due to the low numbers. We also had low numbers of multiple infections with both HPV16 and other HPV genotypes in the original study and chose to not include any such samples in this study.

Finally, most of the smears analysed in this study were originally sampled in the late 1980s to early 2000s, meaning that the isolates we observed may be somewhat different from today’s circulating isolates. However, given the limited mutation rate of HPV types over time^2^, we do not believe this to have substantially influenced our results.

In conclusion, we here describe high-precision lineage assignation and mutation calling for a national population-based study of women with normal smears who later developed cervical cancer in situ or invasive cervical cancer. A1/A2 variant sublineage infections were most common, and the *E7* gene exhibited the least variability among the HPV genes—close to the point of full conservation. The large HPV16 genomic variability would be of interest to study as a possible risk factor in cervical screening.

## Electronic supplementary material

Supplemental Material

## Data Availability

Individual-level data may be shared on request if all legal and ethical requirements are met. Requests should be sent to the corresponding author.
