# Supplementary material for: Human papillomavirus type 16 genomic variation in women with subsequent in situ or invasive cervical cancer: prospective population-based study
Source: Br J Cancer. 2018 Oct 22;119(9):1163–8. doi: 10.1038/s41416-018-0311-7 (PMC6219482; doi:10.1038/s41416-018-0311-7)
Supplement: Supplementary file 1 — Supplemental Material [file 41416_2018_311_MOESM1_ESM.docx]

**Supplementary Table 1. Pools of primers for each PCR reaction.**

| PCR REACTION 1 | PCR REACTION 2 | PCR REACTION 3 | PCR REACTION 4 | PCR REACTION 5 |
| --- | --- | --- | --- | --- |
| HPV16_7_F | HPV16_1_F | HPV16_2_F | HPV16_19_F | HPV16_10_F |
| HPV16_7_R | HPV16_1_R | HPV16_2_R | HPV16_19_R | HPV16_10_R |
| HPV16_9_F | HPV16_3_F | HPV16_4_F | HPV16_22_F | HPV16_12_F |
| HPV16_9_R | HPV16_3_R | HPV16_4_R | HPV16_22_R | HPV16_12_R |
| HPV16_13_F | HPV16_5_F | HPV16_6_F | HPV16_24_F | HPV16_21_F |
| HPV16_13_R | HPV16_5_R | HPV16_6_R | HPV16_24_R | HPV16_21_R |
| HPV16_15_F | HPV16_14_F | HPV16_8_F | HPV16_28_F | HPV16_23_F |
| HPV16_15_R | HPV16_14_R | HPV16_8_R | HPV16_28_R | HPV16_23_R |
| HPV16_15B_R | HPV16_14B_R | HPV16_8B_F | HPV16_28B_R | HPV16_26_F |
| HPV16_17_F | HPV16_16_F | HPV16_8B_R | HPV16_32_F | HPV16_26_R |
| HPV16_17_R | HPV16_16_R | HPV16_11_F | HPV16_32_R | HPV16_30_F |
| HPV16_20_F | HPV16_31_F | HPV16_11_R | HPV16_34_F | HPV16_30_R |
| HPV16_20_R | HPV16_31_R | HPV16_18_F | HPV16_34_R | HPV16_33_F |
| HPV16_20B_F | HPV16_31B_R | HPV16_18_R | HPV16_43_F | HPV16_33_R |
| HPV16_25_F | HPV16_36_F | HPV16_29_F | HPV16_43_R | HPV16_39_F |
| HPV16_25_R | HPV16_36_R | HPV16_29_R | HPV16_45_F | HPV16_39_R |
| PV16_27_F | HPV16_38_F | HPV16_29B_F | HPV16_45_R |  |
| HPV16_27_R | HPV16_38_R | HPV16_37_F |  |  |
| HPV16_35_F | HPV16_42_F | HPV16_37_R |  |  |
| HPV16_35_R | HPV16_42_R | HPV16_40_F |  |  |
| HPV16_41_F | HPV16_46_F | HPV16_40_R |  |  |
| HPV16_41_R | HPV16_46_R |  |  |  |
| HPV16_44_F |  |  |  |  |
| HPV16_44_R |  |  |  |  |
| HPV16_47_F |  |  |  |  |
| HPV16_47_R |  |  |  |  |

**Supplementary Table 2. SNPs classified as novel, by position of the HPV16 genome (relative to reference genome HPV16REF, 7906 bp).**

| SNP | REF | ALT | SNP | REF | ALT | SNP | REF | ALT | SNP | REF | ALT | SNP | REF | ALT | SNP | REF | ALT | SNP | REF | ALT |
| --- | --- | --- | --- | --- | --- | --- | --- | --- | --- | --- | --- | --- | --- | --- | --- | --- | --- | --- | --- | --- |
| 16 | G | T | 1267 | G | A | 1838 | G | C | 3557 | C | T | 4229 | A | G | 5193 | T | G | 6615 | G | A |
| 38 | G | A | 1279 | G | A | 1937 | C | T | 3579 | A | G | 4327 | G | A | 5198 | G | A | 6640 | T | G |
| 53 | G | A | 1317 | T | G | 2093 | G | A | 3590 | A | C | 4411 | G | C | 5247 | A | T | 6738 | G | A |
| 57 | C | T | 1322 | C | T | 2181 | G | A | 3599 | A | C | 4416 | T | C | 5396 | C | G | 6850 | T | C |
| 59 | G | A | 1352 | G | A | 2281 | A | C | 3702 | C | T | 4439 | G | A | 5420 | C | T | 6937 | T | C |
| 78 | A | T | 1358 | G | A | 2356 | G | C | 3712 | G | T | 4447 | G | A | 5437 | C | T | 7113 | C | A |
| 87 | A | T | 1361 | G | A | 2363 | G | A | 3720 | C | T | 4513 | T | G | 5531 | C | T | 7123 | T | C |
| 106 | G | A | 1385 | G | T | 2390 | G | A | 3723 | G | T | 4543 | C | A | 5533 | G | T | 7136 | C | T |
| 121 | G | A | 1388 | G | A | 2407 | G | C | 3955 | G | C | 4556 | C | T | 5549 | C | A | 7187 | T | C |
| 264 | A | T | 1390 | G | A | 2461 | G | A | 3971 | C | T | 4567 | G | A | 5604 | A | C | 7192 | T | G |
| 388 | G | A | 1392 | G | A | 2488 | G | A | 3985 | G | A | 4598 | C | T | 5667 | C | T | 7195 | A | G |
| 471 | A | G | 1393 | G | A | 2583 | T | C | 3998 | G | A | 4608 | T | G | 5671 | C | T | 7261 | T | C |
| 525 | G | A | 1396 | G | C | 2592 | G | A | 4022 | G | A | 4629 | A | T | 5678 | C | T | 7334 | T | A |
| 588 | T | C | 1399 | G | A | 2739 | C | T | 4062 | A | G | 4707 | A | C | 5706 | G | A | 7515 | A | G |
| 739 | A | G | 1464 | T | G | 3006 | A | G | 4066 | T | C | 4727 | C | T | 5746 | T | C | 7519 | A | G |
| 781 | G | a | 1531 | G | A | 3023 | G | A | 4109 | G | A | 4762 | G | A | 5751 | G | A | 7541 | C | A |
| 904 | G | A | 1535 | G | A | 3089 | A | C | 4134 | A | T | 4925 | G | A | 5917 | A | C | 7548 | C | T |
| 928 | G | A | 1542 | T | C | 3170 | A | G | 4160 | T | C | 5000 | C | T | 6081 | C | G | 7597 | T | A |
| 1099 | C | T | 1563 | T | C | 3176 | G | A | 4187 | T | G | 5116 | T | G | 6152 | G | A | 7638 | C | G |
| 1132 | T | A | 1637 | A | C | 3314 | C | A | 4188 | T | G | 5125 | A | G | 6491 | A | T | 7804 | G | T |
| 1160 | G | T | 1678 | G | T | 3529 | C | G | 4221 | C | T | 5189 | A | G | 6516 | C | T | 7852 | G | A |
